# Effect of age and sex on the associations between potential modifiable risk factors and both type 2 diabetes and impaired fasting glycaemia among West African adults

**DOI:** 10.1186/s12889-022-13588-w

**Published:** 2022-06-17

**Authors:** Ayuba Issaka, Adrian J. Cameron, Yin Paradies, William K. Bosu, Yèssito Corine N. Houehanou, Jean B. Kiwallo, Chea S. Wesseh, Dismand S. Houinato, Diarra J. P. Nazoum, Christopher Stevenson

**Affiliations:** 1 School of Health and Social Development, Faculty of Health, Deakin University, Waurn Ponds Campus, Locked Bag 20000, Geelong, VIC 3220 Australia; 2 Alfred Deakin Institute for Citizenship and Globalisation, Faculty of Arts and Education, Deakin University, 221 Burwood Highway, Burwood, VIC 3125 Australia; 3 Baker Heart and Diabetes Institute, 75 Commercial Road, Melbourne, VIC 3004 Australia; 4 West Africa Health Organization, 01 BP 153, Bobo-Dioulasso, Burkina Faso; 5 National School of Senior Technicians Training in Public Health and Epidemiological Surveillance, University of Parakou, Postal Box 122, Parakou, Benin; 6 Directorate of Population Health Protection (DPSP) of the Burkina Faso, Ministry of Health, Ouagadougou, Burkina Faso; 7 Ministry of Health, Republic of Liberia. Congo Town, Monrovia, Liberia; 8 Laboratory of Epidemiology of Chronic and Neurological Diseases (LEMACEN), Faculty of Health Sciences: 01 Postal, University of Abomey Calavi, Box 188, Cotonou, Benin; 9 Former Head of Noncommunicable Diseases, National Directorate of Health, Ministry of Health and Public Hygiene, Bomako, Mali

**Keywords:** T2DM, IFG, Age difference, Sex difference, Risk factors, Associations, West Africa

## Abstract

**Background:**

Type 2 diabetes mellitus (T2DM) is becoming one of the leading causes of morbidity and mortality worldwide, including among Africans. Knowledge of the association between traditional risk factors and both diabetes and pre-diabetes, and whether these differ by age and sex, is important for designing targeted interventions. However, little is known about these associations for African populations.

**Methods:**

The study used data from WHO STEPS surveys, comprising 15,520 participants (6,774 men and 8,746 women) aged 25–64 years, from 5 different West African countries, namely Burkina Faso (4,711), Benin (3,816), Mali (1,772), Liberia (2,594), and Ghana (2,662). T-test and chi-square tests were used to compare differences in the prevalence of traditional risk factors for both sexes. Multinomial logistic regression was conducted to ascertain the relative risks (RR) and 95% confidence intervals (CI) for both T2DM and impaired fasting glucose (IFG) relating to each risk factor, including obesity [defined by BMI, waist circumference (WC), waist-to-hip ratio (WHR), and waist-to-height ratio (WHtR)], high blood pressure (HBP), fruit and vegetable consumption, physical inactivity, alcohol consumption, and smoking. Models for each of these traditional risk factors and interactions with age and sex were fitted.

**Results:**

Factors associated with T2DM and IFG were age, obesity [defined by BMI, WC, WHtR, and WHR], HBP, smoking, physical inactivity, and fruit and vegetable consumption (*p* < 0.05). Analysis of interaction effects showed few significant differences in associations between risk factors and T2DM according to age or sex. Significant interaction with age was observed for HBP*age and T2DM [RR; 1.20, 95% CI: (1.01, 1.42)) (*p* = 0.04)], WHtR*age and T2DM [RR; 1.23, 95% CI: (1.06, 1.44) (*p* = 0.007)] and WHR*age and IFG [RR: 0.79, 95% CI: (0.67, 0.94) (p = 0.006)]. Some interactions with age and sex were observed for the association of alcohol consumption and both IFG and T2DM, but no clear patterns were observed.

**Conclusion:**

The study found that with very few exceptions, associations between traditional risk factors examined and both IFG and T2DM did not vary by age or sex among the West African population. Policies and public health intervention strategies for the prevention of T2DM and IFG should target adults of any age or sex in West Africa.

## Introduction

Type 2 diabetes mellitus (T2DM) is a leading cause of morbidity and mortality globally [1, 2]. Four million people are estimated to die annually from diabetes and its complications worldwide [3], with middle- and low-income countries experiencing the highest burden [2, 4]. In Africa, non-communicable diseases (NCDs), including T2DM, place a significant financial burden on individuals, families, and economies of countries, including direct (e.g., cost of medication, hospital bills, and admission) and indirect costs (e.g., caring for the sick and loss of productivity due to work absenteeism). Projections from 2019 to 2045 suggest a rapid global increase in the prevalence of diabetes, with sub-Saharan Africa (SSA) being the continent recording the highest growth over the period [4], with a 143% increase compared to Western Pacific (31%), South East Asia (74%), Europe (15%), South and Central America (55%) [4].

The progressive increase of the T2DM burden among the African population has been attributed to a rapid increase in urbanisation and food market globalisation that are associated with changes in traditional lifestyle risk factors (e.g., increased obesity, smoking, alcohol consumption, physical inactivity) that are potentially modifiable [5]. However, the association between these traditional risk factors and both T2DM and pre-diabetes can vary considerably by age and sex among different populations [6–10] with significant implications for prevention and treatment strategies [7, 9]. For example, while younger women of childbearing age are more likely to develop diabetes than younger men (due to gestational diabetes), the risk is greater for older men than women [9, 11, 12].

Sex differences in the association between NCDs risk factors and T2DM have been reported in different populations and ethnicities [7, 8, 13, 14] with some of these traditional risk factors having stronger associations with T2DM in men, and others in women. A tri-ethnic prospective study showed that insulin resistance and central obesity among Indian Asians and African Caribbean populations accounted for a twofold greater incidence in women, but not men [15]. Various prospective studies among European populations demonstrated a positive association between body mass index (BMI) and T2DM only in men [6], only in women [16], and in both men and women [7, 8, 10]. Other prospective studies demonstrated risk factors such as high-density lipoprotein cholesterol and physical inactivity during leisure time to be associated with T2DM development in women only [7, 8], while elevated systolic blood pressure, regular smoking, and high daily alcohol intake predicted the development of T2DM in men only [7].

Although the association between T2DM and traditional risk factors can be modified by age and sex in various populations [7, 11], such studies among the African populations are scarce [9, 10]. This is despite the rapidly rising T2DM rates within the continent [4]. Given that sex and age differences in the association between risk factors and T2DM may have implications for both clinical decision making and preventive health strategies, this study assesses whether sex and age modify the associations between potentially modifiable risk factors [including BMI, waist circumference (WC), waist to height ratio (WHtR), waist to hip ratio (WHR), diet, smoking, alcohol, and high blood pressure (HBP)] and both T2DM and pre-diabetes among adults from five West African countries.

## Methods

### Study design, setting, and population

The WHO’s Stepwise Approach to Surveillance (STEPS) survey method was used to collect individual population-level data between 2006 and 2013 from five different West African countries, namely, Benin, Burkina Faso, Ghana, Liberia, and Mali. The STEPS survey is a standardised instrument used to collect information on NCD risk factors in WHO member states [17]. All WHO member states eligible to participate in the survey decide what data to collect based on their needs and interests, as well as available resources and the capacity to implement the survey [17]. The STEPS survey comprises three components, which include data from eight behavioural, biological, and biochemical factors contributing to the burden of NCD, including T2DM. The information is collected through a questionnaire (Step 1); physical examination (Step 2); and biochemical measurements (Step 3). Each country obtained ethical approval from its respective ethics committee. Informed consent was obtained from each participant. Multi-stage cluster sampling was used to randomly select participants [18]. Out of 14 West African countries invited to participate, five countries responded and provided their WHO STEPS data.

### Data processing

The total sample included 16,845 participants before data cleaning. After excluding observations that had incomplete, inconsistent and invalid information, records on 15,520 participants were retained for analysis. To ensure data comparability between countries, only participants aged between 25—64 years were included. The survey response varied across countries from 95.2% (Mali) to 99.4% (Ghana). Detailed information about the study design is available from the reports of participating countries on the WHO STEPS websites [19]. Missing data were generally scant in all countries. We conducted a sensitivity analysis to ascertain the potential effect of data not being missing at random. However, owing to the low proportion of missing values, more complex approaches to missingness such as multiple imputations were not warranted. Details of these sensitivity analyses have previously been reported (see Issaka et al. [20]). Before our sensitivity analysis, we checked for internal consistency and reliability of the data by ensuring that no records with incompatible variable values were included and that all records of variables values agreed with each other. To ensure all values were realistic, we excluded implausible values using the WHO-recommended cut-off values [18]. A measure of HBP was not available for data from Ghana, while hip circumference was not collected in Burkina Faso meaning that the WHR could not be calculated for this country.

### Definitions

All definitions followed the WHO-recommended cut-off values [21]. IFG was defined as 6.1 – < 7.0 mmol/L (110 – 125 mg/dL). T2DM was defined as a fasting plasma glucose reading of ≥ 7.0 mmol/L (> 126 mg/dL). Hypertension was defined as diastolic blood pressure ≥ 90 mmHg and/or systolic blood pressure ≥ 140 mmHg [21]. Participants who reported taking blood pressure lowering medication were classified as having hypertension regardless of their blood pressure measurements in the survey. Normal BMI was defined as 20 – 24.99 kg/m^2^, overweight was defined as BMI of 25 – 29.99 kg/m^2^, and obesity as BMI ≥ 30 kg/m^2^. Elevated WC was defined as ≥ 80 cm for men and ≥ 94 cm for women. Elevated WHR was defined as ≥ 0.90 for men and ≥  0.85 for women. Elevated WHtR was defined as WHtR > 0.5. Fruit and vegetable consumption was defined as inadequate and adequate. Daily smokers were defined as those who currently used tobacco daily, while current alcohol drinkers were defined as those who have drunk alcohol at least once over the last 30 days. Those who drank alcohol every day per week were considered heavy drinkers. Physical activity (low, moderate, or high) was categorised according to self-reported answers to questions from the Global Physical Activity Questionnaire (GPAQ). Owing to the low level of fruit and vegetable consumption in this sub-population, adequate fruit and vegetable consumption was defined as two servings per day instead of the WHO-recommended five servings per day [22]. Among the sociodemographic variables, employment status was dichotomised as either employed or unemployed while educational status was categorised as: none; primary, and secondary/tertiary. Sex was coded as Male = 0 and Female = 1.

### Analysis

Stata 17.0 was utilised for all analyses. All analyses were adjusted for the clustered sampling design used, with data weighted to the age and sex profile of the African standard population [23]. Across all analyses, a p-value of p < 0.05 was considered statistically significant. Variables were described using simple percentages, means (reported as mean ± standard deviation), and frequencies as appropriate. Student t-tests tests were used to compare different group means of male and female participants. Chi-square was used to compare the categorical variables. Multinomial logistic regression was used in a pooled analysis, separately for males and females, to ascertain the relative risks (RR) and 95% confidence intervals (CI) of each risk factor modelled as categorical variables based on established cut-points and the same outcomes. Associations between the traditional risk factors and both T2DM and IFG were assessed in crude analyses, and after adjusting for confounding factors. The confounding factors include age, sex, education, and profession, and were considered as all five countries had data for them. We tested whether the association between these traditional risk factors and both T2DM and IFG varied between males and females and across different age groups by adding interaction terms for sex and age as continuous variables. The *testparm* command in Stata was then used to ascertain the presence of statistically significant interaction simultaneously across the categories of the traditional risk factors and both T2DM and IFG as outcomes.

## Results

Table 1 summarises the socio-demographic characteristics of study participants. Of the 15,520 respondents analysed, 44% were males and 56% were females. The mean and median age of the total sample was 40.4 years and 38 years respectively. The age group with the largest number of participants was between 24 and 34 years (38%) and the lowest was between 55 and 64 (17%) years. The BMI (kg/m^2^) of females was significantly higher than that of males (mean in females = 25.7, mean in males = 23.5, *p* < 0.001) and males were more physically active than females (*p* < 0.001). The smoking and alcohol intakes of males were significantly higher than those of females (smoking, *p* < 0.001 and alcohol consumption, p < 0.001).

**Table 1:** Participant Characteristics

Variable	Participants		
	**Male (*n* = 6774)**	**Female (*n* = 8746)**	**Total (*n* = 15,520)**
Age (years)	40.77 ± 11.8	40.03 ± 11.5	40.36 ± 11.6
BMI (kg/m^2^)	23.47 ± 5.9	25.74 ± 7.9	24.76 ± 7.18
**BMI (%)**			
Normal	4867 (73.7)	4625 (55.4)	9,504 (63.4)
Overweight	1276 (19.3)	2.029 (24.3)	3315 (22.1)
Obese	463 (7.0)	1697 (20.3)	2177 (14.5)
**WC (%)**			
Normal	5850 (87.7)	3233 (39.2)	9084 (60.9)
Elevated	824 (12.3)	5015 (60.8)	5839 (39.1)
**WHR (%)**^a^			
Normal	2595 (58.6)	2059 (34.4)	4655 (44.7)
Elevated	1832 (41.4)	3921 (65.6)	5753 (55.3)
**WHtR (%)**			
Normal	4716 (72.1)	3296 (40.8)	8021 (54.7)
Elevated	1825 (27.9)	4782 (59.2)	6630 (45.3)
**Physical activity (%)**			
Low	1151 (17.2)	2192 (25.2)	3359 (21.8)
Medium	489 (7.3)	852 (9.8)	1348 (8.7)
High	5063 (75.5)	5.643 (65.0)	10,719 (69.5)
**Fruit per week (%)**			
0–13	4738 (79.3)	6200 (79.8)	10,954 (79.6)
14 +	1240 (20.7)	1573 (20.2)	2814 (20.4)
**Vegetable per week (%)**			
0–13	3658 (70.9)	4811 (70.3)	8486 (70.6)
14 +	1502(29.1)	2030 (29.7)	3533 (29.4)
**Current smokers (%)**			
No	5419 (80.1)	8617 (98.6)	14,067 (90.5)
Yes	1348 (19.9)	121 (1.4)	1,471 (9.5)
**Alcohol (%)**			
Every day	696 (10.7)	300 (3.5)	996 (6.6)
5–6 time per week	263 (4.1)	137 (1.6)	400 (2.7)
1-4time per week	1592 (24.6)	1393 (16.4)	2985 (19.9)
< 1 time per week	505 (7.8)	801 (9.4)	1306 (8.7)
Never	3430 (52.9)	5891 (69.1)	9353 (62.2)
**Profession (%)**			
Employed	5654 (83.8)	5522 (63.5)	11,201 (72.4)
Unemployed	1093 (16.2)	3173 (36.5)	4275 (27.6)
**Education (%)**			
None	3068 (45.7)	4953 (57.0)	8028 (52.0)
Primary	1309 (19.5)	1462 (16.8)	2773 (18.0)
Secondary/Tertiary	2344 (34.9)	2279 (26.2)	4638 (30.0)
**Blood Pressure (%)**^b^			
Normal	4251 (73.0)	5053 (73.1)	9333 (73.1)
Hypertension	1568 (27.0)	1856 (26.9)	3429 (26.9)

^a^Burkina Faso excluded

^b^Ghana excluded

The RRs and 95% CIs of the association between the traditional risk factors and both T2DM and IFG are shown in Table 2. Except for alcohol, and fruit and vegetable consumption, all traditional risk factors showed positive associations with T2DM and IFG both in the crude and adjusted analyses. As expected, associations were mostly stronger between the traditional risk factors and T2DM compared to IFG. The analysis shows that the risk of T2DM increases with increasing age. The highest RR associated with T2DM was recorded among 55 to 64 years old [RR: 4.77, 95% CI: (3.77, 6.04)]. In contrast, for IFG the highest RR was recorded among participants who were obese, as defined by BMI [RR: 2.10, 95% CI: (1.70, 2.59)]. Physical inactivity was strongly associated with both T2DM [RR: 2.02 95% CI (1.68 2.42) and IFG [RR: 1.87, 95% CI (1.87, 2.24)] in the adjusted analyses.

**Table 2:** Association between modifiable risk factors and both T2DM and IFG in five countries from West Africa (*n* = 15,520)

Risk Factors	T2DM				IFG			
	**Crude**		**Adjusted**		**Crude**		**Adjusted**	
	**RR (95% CI)**	***P*-value**	**RR (95% CI)**	***P*-value**	**RR (95% CI)**	***P*-value**	**RR (95% CI)**	***P*-value**
**Sex**								
Male	Reference		Reference		Reference		Reference	
Female	1.01 (0.87, 1.19)	0.87	1.06 (0.89, 1.25)	0.51	1.01 (0.86, 1.18)	0.91	0.93 (0.79, 1.10)	0.4
**Age (years)**								
25–34	Reference		Reference		Reference		Reference	
35–44	1.98 (1.55, 2.51)	< 0.001	2.15 (1.69, 2.75)	< 0.001	1.10 (0.90, 1.34)	0.35	1.19 (0.98, 1.46)	0.09
45–54	2.56 (2.00, 3.26)	< 0.001	2.96 (2.31, 3.79)	< 0.001	1.19 (0.96, 1.47)	0.12	1.32 (1.06, 1.65)	0.013
55–64	4.04 (3.21, 5.07)	< 0.001	4.77 (3.77, 6.04)	< 0.001	1.21 (0.97, 1.51)	0.09	1.34 (1.07, 1.69)	0.011
**BMI (kg/m**^**2**^**)**								
Normal	Reference		Reference		Reference		Reference	
Overweight	1.86 (1.54, 2.25)	< 0.001	1.66 (1.36, 2.03)	< 0.001	1.29 (1.06, 1.58)	0.01	1.23 (1.00 1.51)	0.05
Obese	2.54 (2.08, 3.11)	< 0.001	1.96 (1.57, 2.45)	< 0.001	2.29 (1.89, 2.78)	< 0.001	2.10 (1.70, 2.59)	< 0.001
**WC**								
Normal	Reference		Reference		Reference		Reference	
Elevated	2.71 (2.30, 3.20	< 0.001	2.70 (2.20, 3.32)	< 0.001	1.52 (1.30, 1.78)	< 0.001	1.54 (1.27, 1.88)	0.001
**WHtR**								
Normal	Reference		Reference		Reference			
Elevated	2.75 (2.32, 3.27)	< 0.001	2.14 (1.77, 2.59)	< 0.001	1.64 (1.40, 1.93)	< 0.001	1.55 (1.30, 1.85)	< 0.001
**WHR**^a^								
Normal	Reference		Reference		Reference		Reference	
Elevated	1.81(1.50, 2.18)	< 0.001	1.52 (1.24, 1.85)	< 0.001	1.20 (1.00, 1.44)	0.47	1.23 (1.01, 1.49)	0.04
**Physical activity**								
High	Reference		Reference		Reference		Reference	
Medium	2.16 (1.69, 2.78)	< 0.001	1.63 (1.26, 2.12)	< 0.001	2.06 (1.62, 2.62)	< 0.001	1.74 (1.36, 2.23)	< 0.001
Low	2.35 (1.98, 2.80)	< 0.001	2.02 (1.68, 2.42)	< 0.001	2.10 (1.76, 2.50)	< 0.001	1.87 (1.56, 2.24)	< 0.001
**Fruit per week**								
14 +	Reference		Reference		Reference		Reference	
0–13	1.17 (0.93, 1.46)	0.179	1.33 (1.05, 1.67)	0.02	1.21 (0.97, 1.46)	0.09	1.24 (0.99, 1.55)	0.06
**Vegetables per week (%)**								
14 +	Reference		Reference		Reference		Reference	
0–13	0.77 (0.64, 0.94)	0.01	0.85 (0.69, 1.03)	0.1	1.07 (0.87, 1.31)	0.53	1.09 (0.88, 1.34)	0.42
**Current smoker**								
No	Reference		Reference		Reference		Reference	
Yes	1.38 (1.08, 1.77)	0.01	1.71 (1.31, 2.24)	< 0.001	1.01 (0.83, 1.40)	0.57	1.24 (0.94, 1.64)	0.13
**Alcohol**								
Never	Reference		Reference		Reference		Reference	
< 1 time per week	0.45 (0.32, 0.65)	< 0.001	0.40 (0.28, 0.58)	< 0.001	0.61 (0.45, 0.83)	< 0.001	0.56 (0.41, 0.77)	< 0.001
1-4time per week	0.52 (0.41, 0.66)	< 0.001	0.50 (0.39, 0.63)	< 0.001	0.53 (0.42, 0.67)	< 0.001	0.53 (0.42, 0.67)	< 0.001
5–6 times per week	0.67 (0.40, 1.14)	0.14	0.67 (0.40, 1.15)	0.15	0.44 (0.23, 0.84)	0.012	0.46 (0.24, 0.86)	0.02
Every day	0.41 (0.27, 0.62)	< 0.001	0.35 (0.22, 0.54)	< 0.001	0.52 (0.36, 0.76)	< 0.001	0.54 (0.37, 0.79)	0.001
**Blood pressure**^b^								
Normal	Reference		Reference		Reference		Reference	
Hypertension	2.18 (1.83, 2.60)	< 0.001	1.56 (1.29, 1.90)	< 0.001	1.18 (1.00, 1.41)	0.034	1.08 (0.89, 1.30)	0.42

Adjusted for sex, age, education, and profession

^a^Burkina Faso excluded

^b^Ghana excluded

The interactions with both age and sex for the association between the traditional risk factors and T2DM and IFG are presented in Table 3. The associations between most traditional risk factors and both T2DM and IFG did not vary according to either age or sex. However, a statistically significant interaction with age was observed for the associations between hypertension and T2DM, WHtR and T2DM, and WHR and IFG.

**Table 3:** Interactions with age and sex for associations between potential modifiable risk factors and both T2DM and IFG in five West Africa countries (*n* = 15,520)

Risk Factors	T2DM				IFG			
	**Interaction with age**		**Interaction with sex**		**Interaction with age**		**Interaction with sex**	
	**95% CI for RR**	**P-value**	**95% CI for RR**	**P-value**	**95% CI for RR**	**P-value**	**95% CI for RR**	**P-value**
**BMI**								
Normal	Reference		Reference		Reference		Reference	
Overweight	1.08 (0.91, 1.28)	0.37	0.97 (0.64, 1.43)	0.89	0.99 (0.98, 1.01)	0.81	0.96 (0.64, 1.43)	0.83
Obese	1.05 (0.87, 1.26)	0.63	0.68 (0.44, 1.08)	0.1	0.88 (0.73, 1.05)	0.16	0.88 (0.56, 1.36)	0.55
**WC**								
Normal	Reference		Reference		Reference		Reference	
Elevated	1.01 (0.98, 1.31)	0.13	0.79 (0.54, 1.17)	0.24	0.95 (0.82, 1.09)	0.44	0.80 (0.55, 1.19)	0.26
**WHtR**								
Normal	Reference		Reference		Reference		Reference	
Elevated	1.23 (1.06, 1.44)	0.007	1.15 (0.80, 1.67)	0.46	0.95 (0.98, 1.01)	0.5	1.00 (0.71, 1.41)	0.99
**WHR**^a^								
Normal	Reference		Reference		Reference		Reference	
Elevated	1.00 (0.98, 1.02)	0.96	0.99 (0.67, 1.46)	0.94	0.79 (0.67, 0.94)	0.006	0.89 (0.61, 1.30)	0.55
**Physical activity**								
High	Reference		Reference		Reference		Reference	
Medium	0.98 (0.78, 1.21)	0.83	1.32 (0.78, 2.28)	0.31	0.87 (0.70, 1.08)	0.21	0.89 (0.54. 1.47)	0.66
Low	1.02 (0.87, 1.19)	0.85	1.22 (0.84, 1.77)	0.29	0.93 (0.80, 1.09)	0.38	0.85 (0.60, 1.23)	0.39
**Fruit per week**								
14 +	Reference		Reference		Reference		Reference	
0–13	1.01 (0.82, 1.4)	0.93	0.93 (0.58, 1.48)	0.76	1.02 (0.84, 1.24)	0.87	0.81 (0.52, 1.28)	0.36
**Vegetable per week**								
14 +	Reference		Reference		Reference		Reference	
0–13	1.00 (0.82, 1.24)	0.93	0.84 (0.56, 1.25)	0.39	1.02 (0.84, 1.24)	0.87	1.03 (0.67, 1.57)	0.9
**Smoking**								
No	Reference		Reference		Reference		Reference	
Yes	0.86 (0.70, 1.07)	0.18	1.30 (0.59, 2.87)	0.52	1.02 (0.81, 1.29)	0.18	1.02 (0.39, 2.66)	0.97
**Alcohol**								
Never	Reference		Reference		Reference		Reference	
< 1 time per week	1.00 (0.72, 1.38)	0.99	1.64 (0.74, 3.67)	0.38	0.88 (0.66, 1.18)	0.39	0.49 (0.25, 0.90)	0.03
1-4time per week	1.07 (0.87, 1.33)	0.53	0.51 (0.31, 0.85)	0.01	0.81 (0.65, 1.00)	0.04	077 (0.48. 1.23)	0.28
5–6 times per week	0.73 (0.46, 1.16)	0.18	0.31 (0.07, 1.40)	0.21	0.87 (0.50, 1.52)	0.62	0.23 (0.03, 1.88)	0.17
Every day	0.68 (0.46, 1.00)	0.05	0.25 (0.06, 1.08)	0.06	0.73 (0.53, 1.02)	0.07	0.92 (0.40, 2.16)	0.84
**HBP**^b^								
Normal	Reference		Reference		Reference		Reference	
Hypertension	1.20 (1.01, 1.42)	0.04	1.11 (0.77, 1.60)	0.98	1.01 (0.86, 1.19)	0.87	0.92 (0.64, 1.30)	0.18

Note, all results adjusted for sex, age, education, and profession

^a^Burkina Faso excluded

^b^Ghana excluded

## Discussion

To our knowledge, this is the first study to explore the effect of age and sex on the relationship between traditional diabetes risk factors and T2DM and IFG in West African countries. As expected, we found that the associations between all traditional risk factors, and both T2DM and IFG were significant, even after adjusting for age, sex, profession, and education. The general findings on the traditional risk factors were concordant with those of several previous studies among different population groups, including those from Nigeria [10], Australia [24], Asia, and European countries [7, 8, 25, 26]. For most of the traditional risk factors examined, there was no evidence that associations varied according to either age or sex in the current study.

An important finding from the present study was that obesity as measured using BMI or WC was strongly associated with both T2DM and IFG in both sexes and across all age groups, confirming previous studies among populations of African origin [10, 27], and others of Asian and Europid origin [7, 15, 28]. One study by Lasky et al. [9] among Ugandan subjects found a strong, direct relationship between BMI and the presence of T2DM among women only. This difference could be because, in the Lasky et al. study [9], male subjects were primarily lean (defined as BMI < 20 kg/m^2^) whereas, in the current study, male participants ranged from normal BMI to obese. Although some statistically significant interactions with age were observed for the associations between WHtR and T2DM and between WHR and IFG, their relevance in clinical or public health practice may be limited as these measures (i.e., complex ratios) of obesity are not commonly used.

In the present study, in both sexes, the stronger association between WC and WHtR with T2DM (compared to overall body fat as measured by BMI) in the adjusted models reinforces the importance of abdominal adiposity as an independent risk factor for the development of T2DM [29, 30]. Although obesity, as defined by BMI, had the strongest association with IFG, this is a transition state before T2DM, and it may be the case that those classified as obese based on markers of central obesity spend less time in the IFG category [20]. Of note, glycaemic profiles have been shown to differ by sex [13, 31], with studies among populations from Mauritius and Australia finding impaired glucose tolerance to be more common in women (due to the greater glucose load taken relative to body size) and IFG more common in men [13, 31]. The fact that an oral glucose tolerance test (OGTT) was not used for diagnosis of T2DM or pre-diabetes in the current study means that the comparison of results with other studies that did use an OGTT should be interpreted with caution. Moreover, the thresholds used for defining obesity markers are not consistent across studies.

Overall, low physical activity in the present study was found to be associated with around a two-fold higher risk of both T2DM and IFG, among both sexes and age groups. Previous studies among African populations have reported similar findings independent of BMI [32], as have studies from Portugal [33], the United Kingdom, Canada, Australia, and Finland [28, 34]. In a study among European participants, however, low levels of leisure-time physical activity (e.g. swimming, jogging) were associated with incident diabetes among women only [7]. Although the GPAQ used in this study did include an assessment of leisure-time physical activity, levels of leisure-time physical activity are consistently low across African countries [32].

Our finding that hypertension was associated with T2DM and IFG among both sexes is in direct agreement with earlier studies in Kenya [35] and Europe [7]. In various European prospective cohort studies [7, 36], however, a statistically significant association between systolic blood pressure and T2DM was only observed among men. Those findings have been ascribed to the fact that women with hypertension controlled their level of blood pressure better than men [7], implying the importance of awareness and management of HBP among the West African populations [37]**.**

Furthermore, our finding of statistically significant interaction with age for the association between hypertension and T2DM can be ascribed to the evidence that hypertension increases with age and is also concordant with previous studies among different population groups [38, 39]. For example, in a prospective study among the US population [39], Lai et al. [39], showed a positive interaction with age for the association between insulin sensitivity index and incident hypertension. Similar findings were reported among the Chinese population, in a study by Wan et al. [38].

It is unclear, however, in the current study as in previous observational studies [38, 39] if the associations observed are causal. Although a meta-analysis of prospective studies by Emodin et al. [40] postulates that participants with elevated HBP are at increased risk of T2DM, longitudinal studies among populations from the UK [41], China [38], and the US [42], showed that T2DM may be in the causal pathway of hypertension whereas the opposite was not likely. As such, a fine-grained longitudinal study examining the effect of age and the association between hypertension and T2DM is required to ascertain causality among the West African population.

In the current study, moderate drinking of alcohol was found to be protective for T2DM and IFG, which is consistent with previous studies [43]. Heavy alcohol use, on the other hand, has previously been found to be associated with T2DM in both sexes and all age groups [44, 45]. Almost 70% of women in the current sample have never drunk alcohol, with this being due to religious and cultural factors in West Africa [46]. The low level of alcohol use in this study may mean that associations between consumption and glucose status may have little public health relevance in this population.

Smoking in this study was associated with T2DM and IFG and confirms earlier studies among South Africans and other populations [47]. Though the association between smoking and T2DM and IFG did not vary by sex or age in this study, some previous studies among European populations [6, 7] showed positive associations between cigarette smoking and incident diabetes in men only. An association was evident between cigarette smoking and incident diabetes in women however in the large American Nurses' Health Study [16]. The difference in the prevalence of smoking among women in these two studies (much higher in the Nurses Health Study) may explain these findings [6, 7]. As such, the low percentage of female smokers (1.4% of women and 19.9% of men) in the present study means it is challenging to assess sex-specific differences in associations with T2DM and pre-diabetes.

Finally, earlier studies on dietary patterns conducted in urban Ghana [48] and Senegal [49] showed that inadequate fruit and vegetable consumption was associated with an increased risk of T2DM. In this study, lower fruit intake was associated with increased prevalence of T2DM but the association with IFG was not statistically significant. The lower vegetable intake had opposite associations with T2DM and IFG although none were statistically significant. The simple diet recall questions used, limit the ability to generalise from these findings, but the results are consistent with previous evidence that fruit consumption alone is protective against T2DM [50].

### Study strengths and limitations

The current study has several strengths, including the large sample size from five different West African countries. This ensured greater statistical power to detect age and sex interaction effects between potentially modifiable traditional risk factors and both T2DM and IFG. The study, however, has several limitations, which need to be considered when interpreting the results. First, because the study is cross-sectional in design, results do not imply causal relationships between these traditional risk factors and T2DM. Secondly, only the traditional risk factors that were assessed in all five countries were analysed. Therefore, important risk factors, such as high-density lipoprotein cholesterol levels, could not be included in this analysis, though studies have shown this factor to interact with sex [8]. Thirdly, since an OGTT was not used to define diabetes and pre-diabetes, the results may differ from those where this method was used, especially given the sex-specific impact of a glucose load due to differences in body size between males and females [31]. Fourthly, fruit and vegetable consumption measures were not coded as per the WHO guidelines of five servings per day due to the low level of fruit and vegetable consumption in this sub-population and may constitute a limitation when compared to other studies. Lastly, the different years that the survey data were collected may have introduced some bias, however, this is not an important limitation for this study given the focus on associations between risk factors and health outcomes. These limitations notwithstanding, the findings from the current study have important policy implications.

### Policy implications

Since the associations between the traditional risk factors and both T2DM and IFG appear to vary minimally based on age or sex, policies and interventions do not need to be tailored to different West African populations based on age or sex. This is particularly advantageous given the low-income context in West Africa, with population-wide interventions likely to be both more cost-effective and simpler to implement. While, in general, smoking and alcohol are more prevalent among men, obesity and physical inactivity are more prevalent among women in West Africa. Any policies targeting these risk factors should consider socio-cultural factors and beliefs [51]. These may include i) the commonly held belief in much of Africa that being overweight is an outward manifestation of high socioeconomic standing, prosperity, and beauty, as well as good health among females and the preference for central obesity among some affluent men [52, 53]; ii) the fact that physical activity among women is often discouraged in most countries as it is culturally considered to be undesirable and unattractive and associated with a masculine physique [51], and iii) the fact that physical activity is usually not viewed through a health lens for men, but through the lens of sports [54]. Policies should target the persistently low levels of awareness regarding the importance of fruit and vegetable consumption, as well as the globalisation of food markets, particularly concerning alcohol and tobacco industries. Globalisation has been shown to exacerbate the use and increased the ease of access to alcohol and tobacco use among young adults in Africa [55].

## Conclusion

This study found strong associations between traditional risk factors and T2DM and pre-diabetes in a West African population. With very few exceptions, associations were consistent across age and sex meaning that interventions and policies for treatment and prevention of T2DM and IFG may be similar among adults of both sexes in West Africa. Importantly, these findings could aid policymakers, government, non-government bodies, and health professionals in the development of specific guidelines at the individual, community-level educational programs for the prevention and treatment of T2DM.

## Data Availability

The datasets generated and/or analysed during the current study are not publicly available because they belong to third parties but are available from the corresponding author on reasonable request.

## Abbreviations

BMI: Body Mass Index
CI: Confidence Interval
DALY: Disability-Adjusted Life Years
GPAQ: Global Physical Activity Questionnaire
HBP: High Blood Pressure
IDF: International Diabetes Federation
IFG: Impaired Fasting Glucose
NCD: Noncommunicable Disease
OGTT: Oral Glucose Tolerance Tests
RR: Relative Risk
SSA: Sub-Saharan Africa
T2DM: Type 2 Diabetes
WC: Waist Circumference
WHR: Waist to Hip Ratio
WHtR: Waist to Height Ratio
